# Changes in Blood Cells and Complements During Relapse Prevention Therapies for Aquaporin-4 Antibody-Positive Neuromyelitis Optica Spectrum Disorder

**DOI:** 10.3390/ijms27020951

**Published:** 2026-01-18

**Authors:** Hiroshi Kuroda, Kazuo Fujihara, Kimihiko Kaneko, Yoshiki Takai, Yuki Matsumoto, Mizuki Otomo, Naoya Yamazaki, Shu Umezawa, Naoki Yamamoto, Naohiro Sakamoto, Chihiro Namatame, Hirohiko Ono, Shuhei Nishiyama, Toshiyuki Takahashi, Tatsuro Misu, Masashi Aoki

**Affiliations:** 1 Department of Multiple Sclerosis Therapeutics, Fukushima Medical University School of Medicine, Fukushima 960-1295, Japan; 2 Multiple Sclerosis and Neuromyelitis Optica Center, Southern TOHOKU Research Institute for Neuroscience, Koriyama 963-8563, Japan; 3 Department of Neurology, Tohoku University Graduate School of Medicine, Sendai 980-8574, Japan; kimihiko.kaneko.b2@tohoku.ac.jp (K.K.); yoshiki.takai.e6@tohoku.ac.jp (Y.T.); yuki.matsumoto.e5@tohoku.ac.jp (Y.M.); mizuki.otomo.e4@tohoku.ac.jp (M.O.); naoya.yamazaki.c4@tohoku.ac.jp (N.Y.); shu.umezawa.b4@tohoku.ac.jp (S.U.); naoki.yamamoto.r3@dc.tohoku.ac.jp (N.Y.); naohiro.sakamoto.d8@tohoku.ac.jp (N.S.); chihiro.namatame.e6@tohoku.ac.jp (C.N.); hirohiko.ono.b2@tohoku.ac.jp (H.O.); shuhei.nishiyama.b7@tohoku.ac.jp (S.N.); tatsuro.misu.d8@tohoku.ac.jp (T.M.); masashi.aoki.c8@tohoku.ac.jp (M.A.); 4 Department of Neurology, National Hospital Organization Yonezawa National Hospital, Yonezawa 992-1202, Japan; t-toshiyuki@mta.biglobe.ne.jp

**Keywords:** neutrophil, platelet, C3, C4, CH50, satralizumab

## Abstract

In this study, blood cell counts and serum C3, C4, and CH50 values at baseline and after more than 6-month drug use were measured to elucidate changes in blood cells and complements during relapse prevention therapies for aquaporin-4 antibody-positive neuromyelitis optica spectrum disorder (AQP4+ NMOSD). A total of 70 patients with AQP4+ NMOSD (87% female, median age 56 years) were enrolled. They were divided into the following treatment groups: glucocorticoids and/or immunosuppressants (GC/IS, *n* = 22), inebilizumab/rituximab (anti-CD19/20, *n* = 13), satralizumab (anti-IL-6R, *n* = 22), and eculizumab/ravulizumab (anti-C5, *n* = 13). At baseline, the blood counts and complement levels did not differ among the groups. At follow-up, the neutrophil and platelet counts in the anti-IL-6R group decreased from those at baseline (*p* < 0.0001 and *p* < 0.001, respectively). Compared with the GC/IS, anti-CD19/20, and anti-C5 groups, the anti-IL-6R group had lower levels of C3 (*p* < 0.0001, *p* < 0.01, and *p* < 0.05, respectively) and C4 (*p* < 0.0001, *p* < 0.01, *p* < 0.001, respectively). Furthermore, the anti-C5 group had significantly lower CH50 levels than the GC/IS, anti-CD19/20, and anti-IL-6R groups (*p* < 0.0001, *p* < 0.0001, *p* < 0.05, respectively). In addition, the anti-IL-6R group had lower CH50 levels than the GC/IS and anti-CD19/20 groups (*p* < 0.001 and *p* < 0.05, respectively). The present study demonstrated that anti-IL-6R therapy broadly and mildly suppressed the complement system and decreased the neutrophil and platelet counts. It also showed that anti-C5 therapy strongly suppressed total complement activity but did not affect the C3 and C4 levels or blood counts. These findings may have implications for the mode of action of the drugs and the risk of adverse drug reactions, including infections.

## 1. Introduction

Aquaporin-4 antibody-positive neuromyelitis optica spectrum disorder (AQP4+ NMOSD) is an inflammatory disease of the central nervous system. It is characterized by severe optic neuritis, longitudinally extensive transverse myelitis, and brain syndromes, such as area postrema and diencephalic syndromes [1]. The main histopathological finding of AQP4+ NMOSD is massive astrocytic destruction caused by the pathogenic AQP4 antibody, along with an activated complement (complement-mediated cytotoxicity) [2,3,4,5,6]. This antibody is produced by CD19-positive B-cell lineage cells, including plasmablasts, which require interleukin-6 (IL-6) signaling to be differentiated and activated [7,8,9,10]. In fact, C5a, IL-6, and plasmablasts are elevated during relapse of AQP4+ NMOSD [7,11,12,13]. In addition, neutrophil plays a major role in the pathogenesis of AQP4+ NMOSD [14,15,16,17].

Glucocorticoids (GCs) and immunosuppressants (ISs) had demonstrated moderate effectiveness in suppressing AQP4+ NMOSD relapse [18,19]. However, biological drugs targeting complement C5, IL-6R, and CD19/20 have recently shown higher efficacy in preventing relapse of the disease, as indicated by international randomized controlled trials, open-label extension studies, and real-world data [20,21,22,23,24,25,26]. To date, the interactions between a specific molecular targeted therapy and other pathological pathways remain unknown. As regards complement pathway, anti-complement therapy strongly suppresses total complement activity like CH50 [27,28], but the effects of other biotherapies on complements remain to be elucidated. As host defense associated with infection risk, the complement system plays a pivotal role in innate immunity together with phagocytic cells, including neutrophil and macrophage.

In this context, it is crucial to evaluate blood cells and complements during immunotherapies to monitor drug effects and infection risk. The present study aimed to elucidate changes in blood cells and complements during relapse prevention therapies for AQP4+ NMOSD.

## 2. Results

### 2.1. Demographics of the Study Participants

This study enrolled 70 patients with AQP4+ NMOSD [median age, 56 (range 20–80) years, 87% female]. Table 1 presents the characteristics of the patients. The patients were divided into the following treatment groups: GC/IS (*n* = 22: GC/IS combined therapy 13, GC monotherapy 5, IS monotherapy 4), anti-CD19/20 (*n* = 13), anti-IL-6R (*n* = 22), and anti-C5 (*n* = 13). The ages at follow-up blood collection [years, median (range)] were 54.5 (26–80), 60 (20–74), 57 (35–69), and 52 (20–73) years in the GC/IS, anti-CD19/CD20, anti-IL-6R, and anti-C5 groups, respectively. The disease durations [years, median (range)] were 5.5 (0.6–32.8), 5.4 (0.5–32.8), 8.8 (0.4–22.7), and 5.3 (0.7–21.6), respectively. The median values of Expanded Disability Status Scale scores at baseline were 3.0 in the four groups, and the ranges are shown in Table 1.

The intervals between the first injection of biologics and follow-up blood collection [months, median (range)] were 7 (6–48) in the anti-CD19/CD20 group, 7.5 (6–46) in the anti-IL-6R, and 8 (6–55) in the anti-C5. The intervals did not differ among biologics. The doses of oral prednisolone (PSL) [mg/day, median (range)] at baseline were 10 (0–20) in the GC/IS group, 10 (0–20) in anti-CD19/CD20, 10 (0–40) in anti-IL-6R, and 10 (0–60) in anti-C5. At follow-up, the values were 2.5 (0–12) in the GC/IS group, 5 (0–15) in anti-CD19/CD20, 4 (0–15) in anti-IL-6R, and 5 (0–8) in anti-C5, respectively. The doses of PSL did not differ among the groups at baseline and follow-up. No relapses occurred between the initial dose of biologics and the follow-up blood collection. No adverse events related to neutropenia or thrombocytopenia including febrile neutropenia or bleeding complications were observed. With regards to serious adverse events, one patient who was administered rituximab developed bacterial pneumonia and required hospitalization, resulting in rituximab treatment discontinuation.

### 2.2. Decrease in Neutrophil and Platelet Counts During Anti-IL-6R Therapy and Increase in Erythrocyte Counts During Anti-CD19/20 Therapy

**Figure 1:**
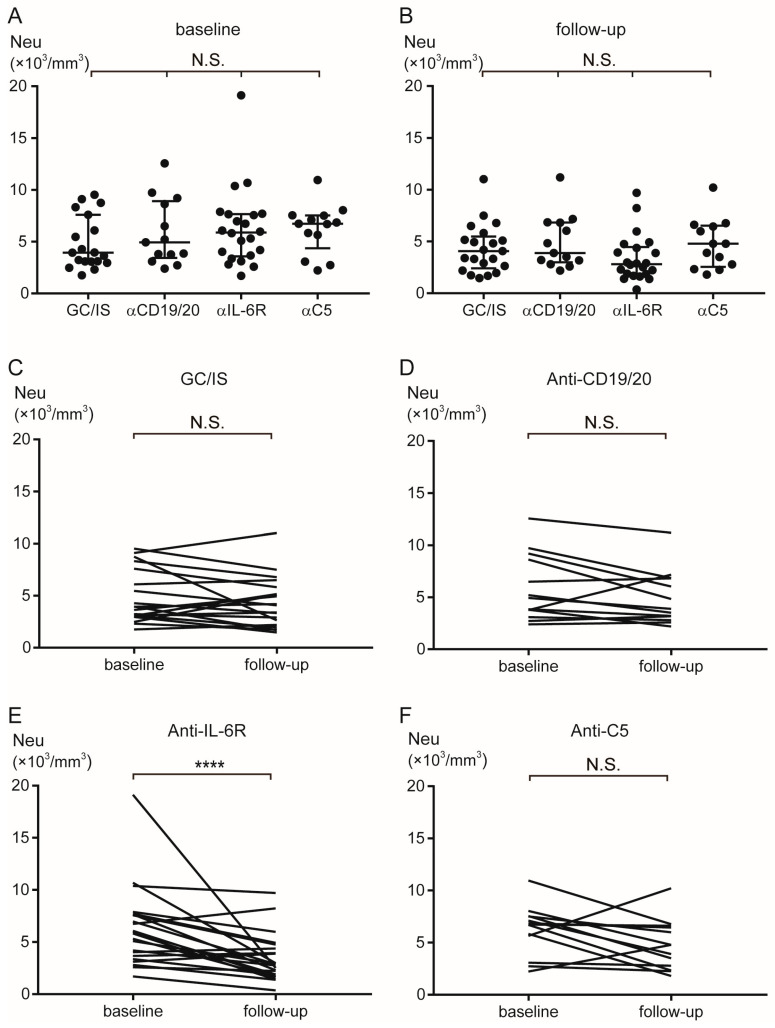
Transition of neutrophil counts during AQP4+ NMOSD treatment. The neutrophil counts did not differ among the groups at baseline (**A**) and at follow-up (**B**). The neutrophil counts did not differ between baseline and follow-up in the GC/IS (**C**), anti-CD19/20 (**D**), and anti-C5 group (**F**) groups. The neutrophil counts decreased from baseline to follow-up in the anti-IL-6R group (**E**). Horizontal bars indicate median and interquartile range. **** *p* < 0.0001. α = anti; AQP4 = aquaporin-4; GC = glucocorticoids; IL-6R = interleukin-6 receptor; IS = immunosuppressants; Neu = neutrophil; NMOSD = neuromyelitis optica spectrum disorder; N.S. = not significant.

**Figure 2:**
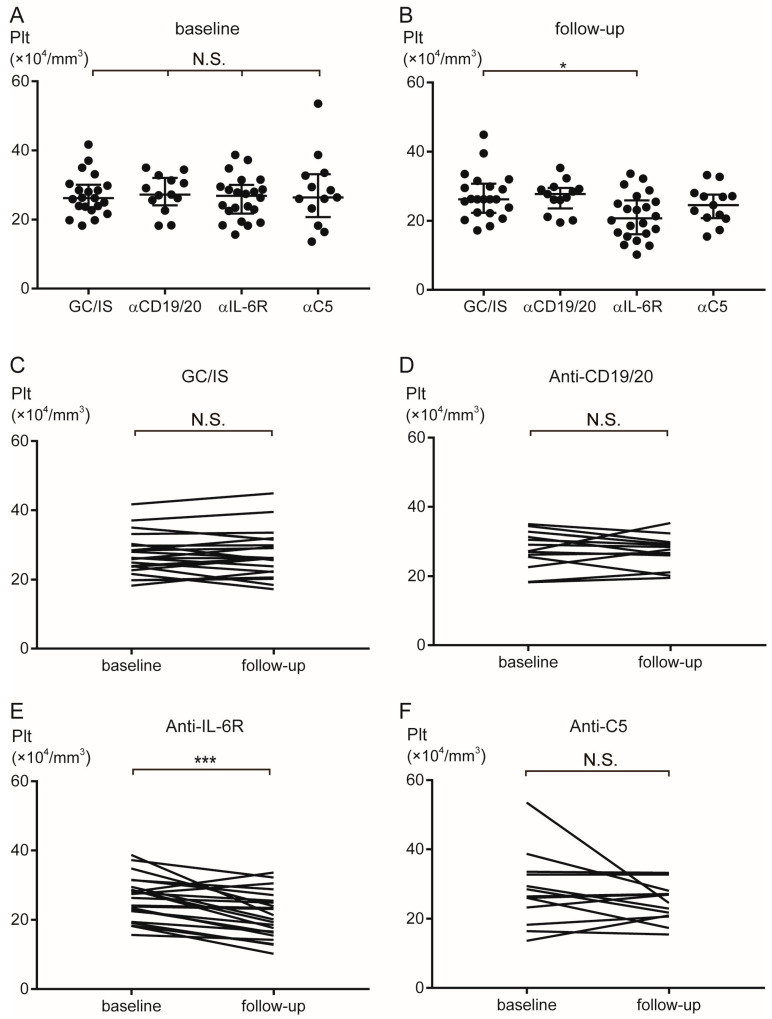
Transition of platelet counts during AQP4+ NMOSD treatment. The platelet counts did not differ among the groups at baseline (**A**). The platelet counts were significantly lower in the ant-IL-6R than in the GC/IS group at follow-up (**B**). The platelet counts did not differ between baseline and follow-up in the GC/IS (**C**), anti-CD19/20 (**D**), and anti-C5 (**F**) groups. The platelet counts decreased from baseline to follow-up in the anti-IL-6R group (**E**). Horizontal bars indicate median and interquartile range. * *p* < 0.05 and *** *p* < 0.001. α = anti; AQP4 = aquaporin-4; GC = glucocorticoids; IL-6R = interleukin-6 receptor; IS = immunosuppressants; NMOSD = neuromyelitis optica spectrum disorder; N.S. = not significant; Plt = platelet.

Table 2 presents the blood cell counts of the patients. At baseline, the blood cell counts did not differ among the groups. However, the anti-IL-6R group showed significantly lower neutrophil counts at follow-up [median 2810/mm^3^ (range 360–9690)] than at baseline [5890 (1700–19,130)] (*p* < 0.0001). Furthermore, the erythrocyte counts in the anti-CD19/20 group at follow-up [median 457 × 10^4^/mm^3^ (range 395–506)] were higher than those at baseline [413 (370–476)] (*p* < 0.001). Moreover, the platelet counts in the anti-IL-6R group were lower at follow-up [median 20.7 × 10^4^/mm^3^ (range 10.2–33.6)] than at baseline [26.9 (15.6–38.7)] (*p* < 0.001). Figure 1 and Figure 2 (neutrocyte and platelet, respectively) depict the comparisons between groups and the transitions from baseline to follow-up.

### 2.3. Decrease in CH50 During Anti-C5 and Anti-IL-6R Therapy and in C3 and C4 During Anti-IL-6R Therapy

**Figure 3:**
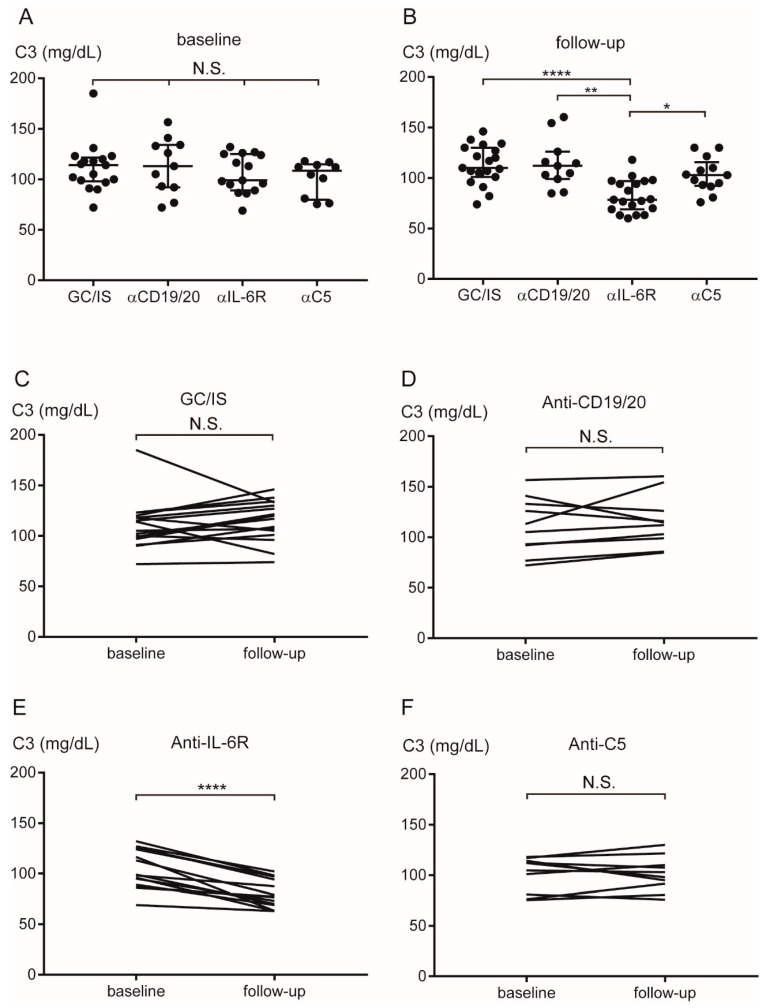
Transition of serum C3 levels during AQP4+ NMOSD treatment. The C3 levels did not differ among the groups at baseline (**A**). The C3 levels were significantly lower in the anti-IL-6R than in the GC/IS, anti-CD19/20, and anti-C5 groups at follow-up (**B**). The C3 levels did not differ between baseline and follow-up in the GC/IS (**C**), anti-CD19/20 (**D**), and anti-C5 (**F**) groups. The C3 levels decreased from baseline to follow-up in the anti-IL-6R group (**E**). Horizontal bars indicate median and interquartile range. * *p* < 0.05, ** *p* < 0.01, and **** *p* < 0.0001. α = anti; AQP4 = aquaporin-4; GC = glucocorticoid; IL-6 = interleukin-6; IS = immunosuppressant; NMOSD = neuromyelitis optica spectrum disorder; N.S. = not significant.

**Figure 4:**
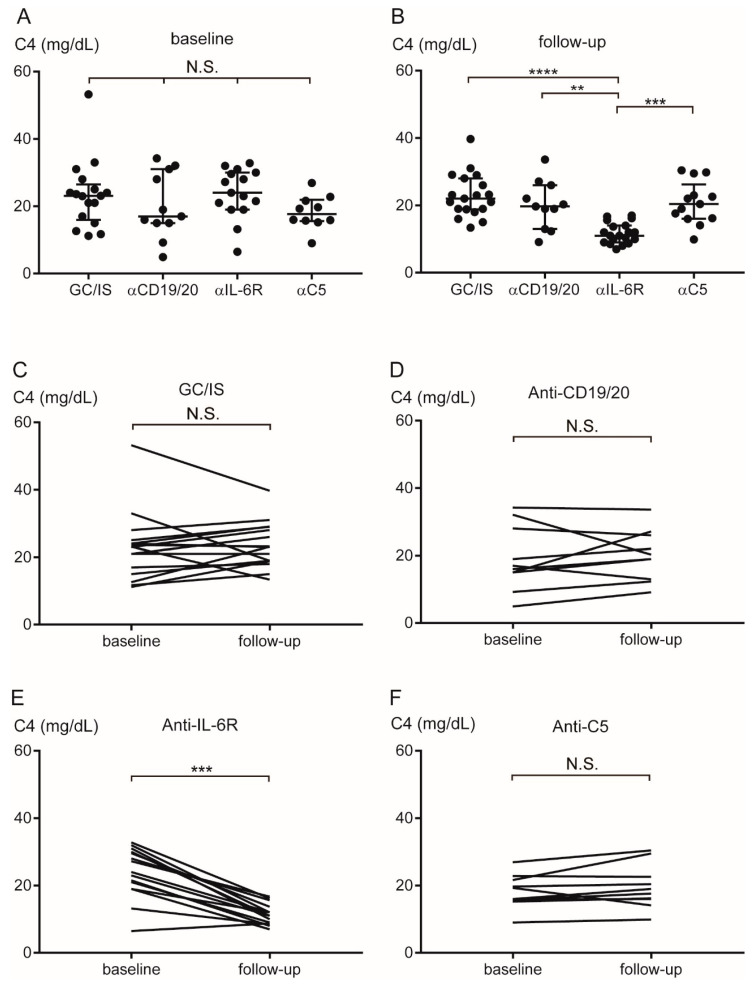
Transition of serum C4 levels during AQP4+ NMOSD treatment. The C4 levels did not differ among the groups at baseline (**A**). The C4 levels were significantly lower in the anti-IL-6R than in the GC/IS, anti-CD19/20, and anti-C5 groups at follow-up (**B**). The C4 levels did not differ between baseline and follow-up in the GC/IS (**C**), anti-CD19/20 (**D**), and anti-C5 (**F**) groups. The C4 levels decreased from baseline to follow-up in the anti-IL-6R group (**E**). Horizontal bars indicate median and interquartile range. ** *p* < 0.01, *** *p* < 0.001, and **** *p* < 0.0001. α = anti; AQP4 = aquaporin-4; GC = glucocorticoid; IL-6R = interleukin-6 receptor; IS = immunosuppressant; NMOSD = neuromyelitis optica spectrum disorder; N.S. = not significant.

Table 3 presents the C3, C4, and CH50 values of the patients. Whereas the C3 levels did not differ among the groups (Figure 3A) at baseline, the C3 levels in the antiIL-6R group [median 78.5 mg/dL (range 60–118)] at follow-up were significantly lower than those in the GC/IS [110 (74–146)], anti-CD19/20 [108 (85–160)], and anti-C5 [103 (76–130)] groups (*p* < 0.0001, *p* < 0.01, and *p* < 0.05, respectively) (Figure 3B). The C3 levels in the GC/IS, anti-CD19/20, anti-C5 groups did not differ between baseline and follow-up (Figure 3C,D,F). The C3 levels in the anti-IL-6R group significantly decreased at follow-up (*p* < 0.0001) (Figure 3E). At baseline, the C4 levels did not differ among the groups (Figure 4A); however, these levels were significantly lower in the anti-IL-6R group [median 11 mg/dL (range 7–17)] than in the GC/IS [22 (13.4–39.7)], anti-CD19/20 [19.7 (9.1–33.6)], and anti-C5 [20.4 (9.9–30.4)] groups at follow-up (*p* < 0.0001, *p* < 0.01, and *p* < 0.001, respectively) (Figure 4B). The C4 levels in the GC/IS, anti-CD19/20, and anti-C5 groups did not differ between baseline and follow-up (Figure 4C,D,F). The C4 levels in the anti-IL-6R group significantly decreased at follow-up (*p* < 0.001) (Figure 4E).

**Figure 5:**
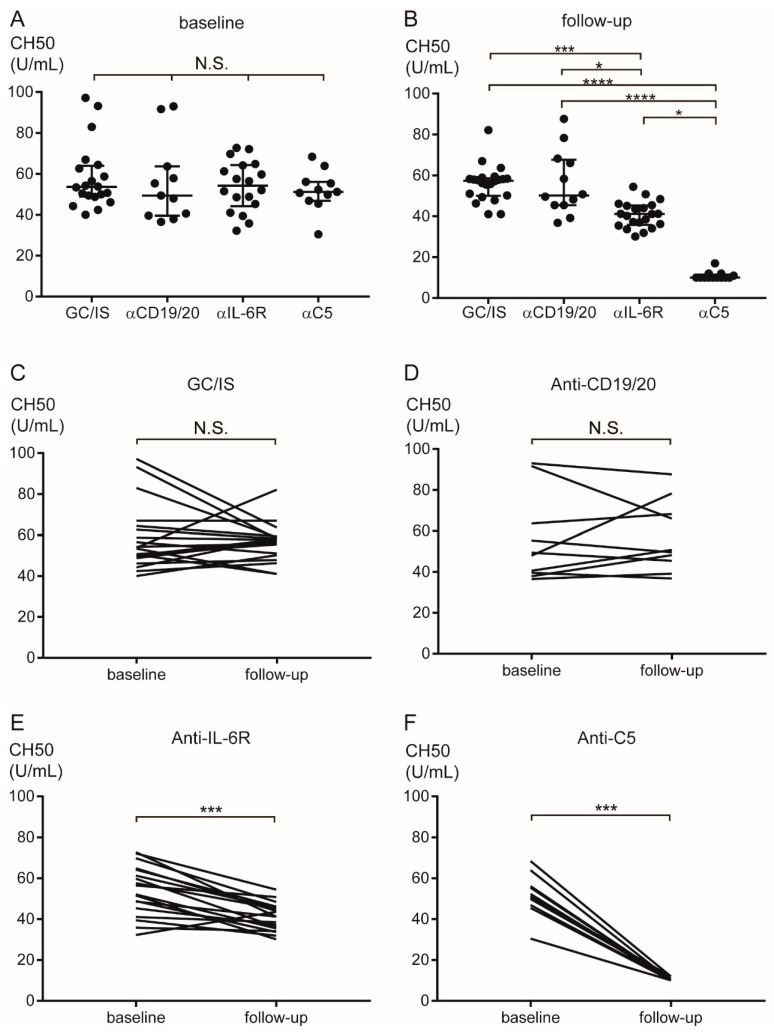
Transition of CH50 values during AQP4+ NMOSD treatment. The CH50 values did not differ among the groups at baseline (**A**). The CH50 values were significantly lower in the anti-C5 than in the GC/IS, anti-CD19/20, and anti-IL-6R groups, and those in the anti-IL-6R group were significantly lower than those in the GC/IS and anti-CD19/20 groups at follow-up (**B**). The CH50 values did not differ between baseline and follow-up in the GC/IS (**C**) and anti-CD19/20 (**D**) groups. The CH50 values decreased from baseline to follow-up in the anti-IL-6R (**E**) and anti-C5 groups (**F**). Horizontal bars indicate median and interquartile range. * *p* < 0.05, *** *p* < 0.001, and **** *p* < 0.0001. α = anti; AQP4 = aquaporin-4; GC = glucocorticoid; IL-6R = interleukin-6 receptor; IS = immunosuppressant; NMOSD = neuromyelitis optica spectrum disorder; N.S. = not significant.

Although the CH50 values at baseline did not differ among the groups (Figure 5A), the CH50 values at follow-up were significantly lower in the anti-C5 group [median 10 U/mL (range 10–17)] than in the GC/IS [57.4 (41–82.1)], anti-CD19/20 [50.8 (36.8–87.6)], and anti-IL-6R [41.1 (30.1–54.4)] groups (*p* < 0.0001, *p* < 0.0001, and *p* < 0.05, respectively). Furthermore, the CH50 values were lower in the anti-IL-6R group than in the GC/IS and anti-CD19/20 groups (*p* < 0.001 and *p* < 0.05, respectively) (Figure 5B). The CH50 values in the GC/IS and anti-CD19/20 groups did not differ between baseline and follow-up (Figure 5C,D). The CH50 values in the anti-IL-6R and anti-C5 group significantly decreased at follow-up (*p* < 0.001 and *p* < 0.001, respectively) (Figure 5E,F).

## 3. Discussion

The present study demonstrated that the anti-IL-6R therapy for AQP4+ NMOSD broadly and mildly suppressed the complement system and decreased neutrophil and platelet counts, whereas the anti-C5 therapy strongly suppressed total complement activity but did not affect the C3 and C4 levels or blood cell counts.

IL-6 is a proinflammatory cytokine that exerts pleiotropic effects on various cells, including thrombopoiesis through stimulation on megakaryocytes and hepatocytes [29,30] and the production of complement proteins from hepatocytes [31], whereas effects on neutrophil is controversial [32]. The activation of IL-6 signal plays a major role in the pathogenesis of AQP4+ NMOSD as well as rheumatoid arthritis (RA) [7,10,33]. Clinical studies of RA have reported that tocilizumab, another anti-IL-6R monoclonal, reduced the C3, C4, and CH50 values [34,35]. The present study confirmed that IL-6 signal blockage reduces C3, C4, and CH50 values. Additionally, it has been reported that the classical pathway is activated through the binding of C-reactive protein to C1q [36]. Considering that CRP production is strongly suppressed under satralizumab treatment, the suppression of this antibody-independent activation of classical pathway might be another possible mechanism of CH50 reduction. With regards to the association between blood cell counts and IL-6 signal, increases in the neutrocyte–lymphocyte ratio (NLR) and platelet–lymphocyte ratios (PLRs) were reported in patients with active RA [37]. However, the ratios decreased after treatment with anti-rheumatic disease-modifying drugs [38,39]. Higher neutrophil counts and NLR were reported in patients with AQP4+ NMOSD than in healthy controls (HCs) or those with multiple sclerosis (MS) [40,41]. In addition, the neutrophils from patients with AQP4+ NMOSD showed an activated phenotype (an increased surface expression of Toll-like receptor 2 and formyl peptide receptor 1) compared with HCs, a compromised functionality (reduced adhesion and migratory capacity as well as decreased reduced production of reactive oxygen species and degranulation) compared with patients with MS [42], and an increased survival capacity in response to phorbol 12-myristate 13-acetate compared with HCs [43]. A decrease in neutrophil counts was also reported in patients with tocilizumab-treated AQP4+ NMOSD, and the study showed the upregulation of neutrophil activation-related genes at baseline and the downregulation of the genes after tocilizumab treatment [44]. Therefore, the anti-IL-6R therapy suppressed neutrophils both in quantitative and qualitative aspects. Regarding satralizumab, the decreases in neutrophils, platelets, and complements have been mentioned briefly in the prescribing information, the clinical trials, and the post-marketing surveillance as adverse reactions [20,23,24,25,45,46]. The present study confirmed these phenomena as real-world data of satralizumab therapy. Although the suppression of the complement pathway and neutrophils is suitable for relapse prevention of AQP4+ NMOSD, there is a high risk of infection in such conditions. Thus, close monitoring of complement activity and neutrophil counts is crucial in patients with AQP4+ NMOSD. The infection rates with long-term satralizumab treatment in the open-label study, SAkuraMoon, and in a post-marketing setting did not increase over time, and concomitant IS use, comorbidities, Expanded Disability Status Scale score ≥ 4.0, and age > 65 years were potential confounders of sepsis [47]. Moreover, a decrease in platelet counts may lead to a bleeding tendency if it progresses to thrombocytopenia, although such a severe condition has not been reported in tocilizumab-treated RA patients [48]. This study demonstrated that decreases in neutrophil and platelet counts as well as C3 and C4 levels were specifically observed in patients treated with satralizumab, regardless of concomitant GC/IS use. If the phenomenon is specific for satralizumab, longitudinal measurements of these parameters will be useful for monitoring drug efficacy and safety.

The present study demonstrated that anti-C5 therapy strongly suppressed total complement activity but did not influence the C3 and C4 levels or blood cell counts. These results indicate that host defense functions such as opsonization and phagocytosis are intact under the treatment. This study also showed that GC/IS and B-cell-depleting therapies exert no effect on complement activity or total blood cell counts, except for increases in erythrocyte counts following B-cell–depleting therapy. The B-cell-depleting therapy is reportedly effective for immune-mediated anemia, such as immune-mediated pure red cell aplasia or lupus-related anemia [49,50]. However, the baseline erythrocyte counts in the anti-CD19/20 group were within normal range, and no hemolytic signs were found in the patients. Therefore, further investigations on erythrocyte counts in patients with AQP4+ NMOSD receiving B-cell-depleting therapies are warranted. Although this study showed that GC/IS therapy exerts no effect on blood cell counts, the pooled data from GC and IS might have masked the contradictory effects on blood cell counts of the drugs. In particular, GCs are known to induce neutrophilia and can increase platelet counts, whereas many immunosuppressants carry a known risk of myelosuppression, including neutropenia and thrombocytopenia.

Our study has limitations. First, this was a small-scale study with short-term follow-up. This highlights the need to confirm our findings in larger-scale studies with longer follow-up periods. Second, quantitative data, such as C3, C4, and blood counts, were mainly analyzed; however, the functions of blood cells and complement should be analyzed further in future studies, and such data would be useful to further elucidate immunological changes induced by relapse prevention therapies for AQP4+ NMOSD.

## 4. Patients and Methods

### 4.1. Selection of Patients

This study was a multicenter observational study. The clinical records and laboratory data of patients with AQP4+ NMOSD were retrospectively reviewed. The patients were categorized into four treatment groups: GC/IS, anti-CD19/20, anti-IL-6R, and anti-C5. Patients who received continuous drug administration for more than 6 months, with blood tests and serum complement measurement, were eligible for this study, as it takes time for each treatment to exert sufficient biological effects. In patients with multiple blood sampling after 6-month drug use, we adopted the first blood sampling as follow-up data.

### 4.2. Standard Protocol Approval, Registration, and Patient Consent

This study was approved by the Ethics Committee of Southern TOHOKU Research Institute for Neuroscience and the Ethics Committee of Tohoku University School of Medicine. Written informed consent was obtained from the patients or informed consent was obtained in the form of opt-out on the institute’s website.

### 4.3. Analyses of Blood Cell Counts and Serum Complement Parameters

Data of peripheral blood cell counts (neutrocyte, lymphocyte, erythrocyte, and platelet) and serum C3, C4, and CH50 levels were collected. Data from blood drawing conducted within 2 weeks after plasma exchange or under infectious conditions were excluded. The methods and reference values for individual serum complement parameters were as follows: C3 [turbidimetric immunoassay, 86.0–160.0 mg/dL], C4 [turbidimetric immunoassay, 15.0–45.0 mg/dL], and CH50 [liposome-based immunoassay, 31.0–48.0 U/mL]. The values in the treatment groups were compared at baseline and after more than 6 months of drug use.

### 4.4. Statistical Analysis

Statistical analyses were conducted using PRISM 7.04 (GraphPad Software, Boston, MA, USA). The Kruskal–Wallis test was employed to compare values between the groups. If a difference was significant based on the test, multiple-comparison post hoc analyses were conducted using Dunn’s test. The Wilcoxon signed-rank test was employed to compare values at baseline and at follow-up. An adjusted *p* < 0.05 was considered to indicate statistical significance.

## 5. Conclusions

This study demonstrated that the anti-IL-6R therapy broadly and mildly suppressed the complement system and decreased neutrophil and platelet counts. Meanwhile, the anti-C5 therapy strongly suppressed total complement activity but did not influence the C3 and C4 levels or blood cell counts. These results may have implications for the mode of action of the drugs and the risk of infections.

## Figures and Tables

**Table 1:** Demographic data of the patients.

	GC/IS Group (*n* = 22)	Anti-CD19/20 Group (*n* = 13)	Anti-IL-6R Group (*n* = 22)	Anti-C5 Group (*n* = 13)
Female sex (%)	82	77	91	92
Age, years, median (range)	54.5 (26–80)	60 (20–74)	57 (35–69)	52 (20–73)
AQP4 antibody-positive, %	100	100	100	100
Disease duration, years, median (range)	6.9 (0.6–32.8)	5.4 (0.5–32.8)	8.8 (0.4–22.7)	5.3 (0.7–21.6)
Baseline EDSS, median (range)	3.0 (1.0–8.0)	3.0 (1.0–7.0)	3.0 (1.0–7.0)	3.0 (2.0–7.0)
Autoimmune complication	CS 1, RA 1, SjS 4	SjS 1	GD 2, RA 1, SjS 3, SLE 1	GD 1, RA 2, SAR 1, SjS 4
Comorbidities potentially affecting blood counts	IDA 1, ITP 1	DVT 1, HPA 1, IDA 1, ITP 1, MT 1	DVT 1, MT 2	DVT 1, MT 1
Treatment just before biologics	N.A.	GC + IS 6, GC 7	GC + IS 9, GC 10, IS 3	GC + IS 1, GC 12
Interval between the first injection of biologics and follow-up, months, median (range)	N.A.	7 (6–48)	7.5 (6–46)	8 (6–55)
Dose of oral PSL, mg/day, median (range)				
Baseline	7.5 (0–50)	10 (0–20)	10 (0–40)	10 (0–60)
Follow-up	5 (0–15)	5 (0–15)	4 (0–15)	5 (0–8)
Concomitant IS, number				
Baseline	AZA 1, TAC 7	AZA 3, TAC 3	AZA 3, TAC 6	AZA 1, TAC 0
Follow-up	AZA 4, TAC 13	AZA 3, TAC 2	AZA 1, TAC 4	AZA 1, TAC 0

AQP4 = aquaporin-4, AZA = azathioprine, CS = chronic thyroiditis, EDSS = Expanded Disability Status Scale, GC = glucocorticoids, GD = Graves’ disease, HPA = hemophilia A, IDA = iron-deficiency anemia, IL-6R = interleukin-6 receptor, IS = immunosuppressants, ITP = idiopathic thrombocytopenic purpura, MT = malignant tumor, N.A. = not applicable, PSL = prednisolone, RA = rheumatoid arthritis, SAR = sarcoidosis, SjS = Sjogren syndrome, SLE = systemic lupus erythematosus, TAC = tacrolimus.

**Table 2:** Blood counts of the patients.

	Neutrophil (/mm^3^)	Lymphocyte (/mm^3^)	Erythrocyte (×10^4^/mm^3^)	Platelet (×10^4^/mm^3^)
GC/IS group				
Baseline	3785 (1760–9530)	1615 (830–2810)	452 (401–521)	27.1 (18.2–37)
Follow-up	3740 (1470–7500)	1780 (620–3270)	439 (366–544)	26.2 (17.2–39.5)
Anti-CD19/20 group				
Baseline	4940 (2400–12,560)	1570 (540–2370)	413 (370–476)	27.2 (18.2–35)
Follow-up	3880 (2190–11,190)	1500 (670–3220)	457 *** (395–506)	27.7 (19.5–35.3)
Anti-IL-6R group				
Baseline	5890 (1700–19,130)	1115 (370–2920)	448 (358–562)	26.9 (15.6–38.7)
Follow-up	2810 **** (360–9690)	1190 (360–2540)	450 (359–515)	20.7 *** (10.2–33.6)
Anti-C5 group				
Baseline	6740 (2220–10,940)	1070 (540–4130)	392 (292–487)	26.4 (13.6–53.5)
Follow-up	4780 (1810–10,200)	1210 (510–2750)	441 (286–486)	24.5 (15.4–33.2)

Values are expressed as median (range). *** *p* < 0.001 and **** *p* < 0.0001 (difference between values at baseline and those at follow-up). GC = glucocorticoid, IL-6R = interleukin-6 receptor, IS = immunosuppressant.

**Table 3:** Serum C3, C4, and CH50 values of the patients.

	C3 (mg/dL)	C4 (mg/dL)	CH50 (U/mL)
GC/IS group			
Baseline	114 (72–185)	23.1 (11.2–53.2)	53.6 (40–97.2)
Follow-up	110 (74–146)	22 (13.4–39.7)	57.4 (41–82.1)
Anti-CD19/20 group			
Baseline	113 (72–156.5)	17 (4.9–34.2)	49.4 (36.5–93)
Follow-up	112 (84.8–160.3)	19.7 (9.1–33.6)	50.2 (36.8–87.6)
Anti-IL-6R group			
Baseline	99 (69–132)	24 (6.5–32.8)	54.2 (32.2–72.7)
Follow-up	78.5 **** (60–118)	11 *** (7–17)	41.1 *** (30.1–54.4)
Anti-C5 group			
Baseline	108.4 (75.5–118)	17.7 (9–26.9)	51.2 (30.4–68.4)
Follow-up	102.8 (76–130)	20.4 (9.9–30.4)	10 *** (10–17)

Values are expressed as median (range). *** *p* < 0.001 and **** *p* < 0.0001 (difference between values at baseline and those at follow-up). GC = glucocorticoid, IL-6R = interleukin-6 receptor, IS = immunosuppressant.

## Data Availability

The data presented in this study are available on request from the corresponding author because the data are not publicly available due to privacy.
